# Complete Coding Sequence of a Lineage AY.122 SARS-CoV-2 Virus Strain Detected in Kazakhstan

**DOI:** 10.1128/mra.00301-23

**Published:** 2023-06-13

**Authors:** Bekbolat Usserbayev, Yergali Abduraimov, Nurlan Kozhabergenov, Aibarys Melisbek, Meirzhan Shirinbekov, Manar Smagul, Gaukhar Nusupbaуeva, Aziz Nakhanov, Yerbol Burashev

**Affiliations:** a Research Institute for Biological Safety Problems, Gvardeyskiy, Kazakhstan; b Scientific and Practical Center for Sanitary and Epidemiological Expertise and Monitoring Branch of the Republican State Enterprise on the Right of Economic Use, National Center for Public Health of the Ministry of Health of the Republic of Kazakhstan, Almaty, Kazakhstan; c Faculty of Biology and Biotechnology, Al-Farabi Kazakh National University, Almaty, Kazakhstan; Queens College Department of Biology

## Abstract

We describe the coding-complete genome sequence of a strain of severe acute respiratory syndrome coronavirus 2 (SARS-CoV-2) obtained from a patient with symptoms of coronavirus disease 2019 (COVID-19), detected in the Republic of Kazakhstan. According to the Pangolin COVID-19 database, the studied strain, SARS-CoV-2/Human/KAZ/Delta-020/2021, belongs to lineage AY.122 and consists of 29,840 nucleotides.

## ANNOUNCEMENT

Coronaviruses (family *Coronaviridae*, order Nidovirales) are RNA viruses that contain single-stranded positive RNA (+ssRNA) as genetic material and range from 26.4 kb to 31.7 kb long (1–3). Currently, the subfamily *Orthocoronavirinae* of the family *Coronaviridae* is divided into 4 genera: *Alphacoronavirus*, *Betacoronavirus*, *Deltacoronavirus*, and *Gammacoronavirus* (https://ictv.global/taxonomy). Until the second decade of the 21st century, mankind faced 6 different pathogens belonging to the subfamily *Orthocoronavirinae*: *Human coronavirus 229E* (HCoV-229E), *Human coronavirus NL63* (HCoV-NL63), *Betacoronavirus 1*, *Human coronavirus HKU1* (HCoV-HKU1), *Severe acute respiratory syndrome coronavirus 1* (SARS-CoV), and *Middle East respiratory syndrome–related coronavirus* (MERS-CoV) (4–8).

An outbreak of a seventh new type of human coronavirus (SARS-CoV-2) was detected in December 2019 in Wuhan, Hubei Province, People’s Republic of China (PRC), and since then, there has been no decrease in the number of infections in the world, resulting in more than 765,222,932 confirmed cases and about 6,921,614 million deaths, as of 3 May 2023 (https://covid19.who.int/) (9). For the first time, SARS-CoV-2 was registered in the territory of the Republic of Kazakhstan (RK) in mid-March 2020 (10, 11). According to WHO data, as of 3 May 2023, more than 1.5 million laboratory-confirmed cases of infection and 19,072 deaths had been detected in the Republic of Kazakhstan (https://covid19.who.int/).

Through the research efforts of many countries to sequence the entire SARS-CoV-2 genome, it has become possible to monitor the spread of different variants of the virus around the world. As of May 2023, about 15.5 million complete genome sequences of SARS-CoV-2 had been deposited in the GISAID database (https://gisaid.org/).

Here, we report the complete coding sequence of the genome of one strain of SARS-CoV-2. The studied strain was obtained from the Scientific and Practical Center for Sanitary and Epidemiological Expertise and Monitoring branch of the Ministry of Health of the Republic of Kazakhstan. Molecular genetic studies were carried out to obtain complete genomic nucleotide sequences of the studied strain of the SARS-CoV-2 virus. The full molecular genetic research design is presented in our previous work (11, 12). To obtain the complete genomic nucleotide sequence of SARS-CoV-2, 65 pairs of sequencing primers were developed, with an overlap of about 100 bp (https://zenodo.org/record/7264509#.ZC-YI3bP1hE). The collected coding-complete genome of strain SARS-CoV-2/Human/KAZ/Delta-020/2021 has a length of 29,840 nucleotides, and the query coverage amounted to 99% of the genome, with a GC content of 38%. To determine the virus strain lineage, the obtained sequences were analyzed using the Pangolin COVID-19 database (https://pangolin.cog-uk.io/) and determined to belong to lineage AY.122. To identify various mutations (amino acid and nucleotide) of the studied strain, the sequences were compared with the reference strain Wuhan-Hu-1 (GenBank accession number NC_045512). The comparison was performed using the COVID-19 genome annotation tool (http://giorgilab.unibo.it/coronannotator/), and the results are presented in Table 1.

**TABLE 1:** Mutations of strain SARS-CoV-2/Human/KAZ/Delta-020/2021 compared to the Wuhan-Hu-1 reference sequence^*a*^

Protein	Data for strain:				Amino acid change
	Wuhan-Hu-1		SARS-CoV-2/Human/KAZ/Delta-020/2021		
	Position	Variant	Variant	Position	
5′ UTR^*b*^	210	G	T	184	210
	241	C	T	215	241
ORF1ab	1048	G	T	1022	K81N^*c*^
	1899	G	T	1873	R365L
	3037	C	T	3011	F106F
	4181	G	T	4155	A488S
	6402	C	T	6376	P1228L
	7124	C	T	7098	P1469S
	8986	C	T	8960	D144D
	9053	G	T	9027	V167L
	10029	C	T	10003	T492I
	11201	A	G	11175	T77A
	11332	A	G	11306	V120V
	14408	C	T	14382	P314L
	15451	G	A	15425	G662S
	16466	C	T	16440	P77L
	18271	G	A	18245	E78K
	18337	G	T	18311	A100S
	19220	C	T	19194	A394V
Spike	21618	C	G	21592	T19R
	21766	A		21739	I68
	21987	G	A	21961	G142D
	22185	C	T	22159	T208M
	22407	A	T	22381	N282I
	22917	T	G	22891	L452R
	22995	C	A	22969	T478K
	23403	A	G	23377	D614G
	23604	C	G	23578	P681R
	24410	G	A	24384	D950N
ORF3a	25469	C	T	25443	S26L
Membrane	26767	T	C	26741	I82T
ORF7	27527	C	T	27501	P45L
	27638	T	C	27612	V82A
	27752	C	T	27726	T120I
	27874	C	T	27848	T40I
ORF8	27919	T	C	27893	I9T
	28251	T	C	28225	F120L
	28253	C	A	28227	F120L
	28255	T	A	28229	I121N
	28258	A	G	28232	*122*^d^
Nucleocapsid	28461	A	G	28435	D63G
	28881	G	T	28855	R203M
	28916	G	T	28890	G215C
	29236	C	T	29210	G321G
	29402	G	T	29376	D377Y
3′ UTR	29742	G	T	29716	29742

a *Severe acute respiratory syndrome coronavirus 2* strain Wuhan-Hu-1, complete genome sequence (GenBank accession number NC_045512).

b UTR, untranslated region.

c K81N, the K-to-N change at position 81.

d *122*, the studied strain according to the Pangolin database belongs to the AY.122 lineage.

**FIG 1:**
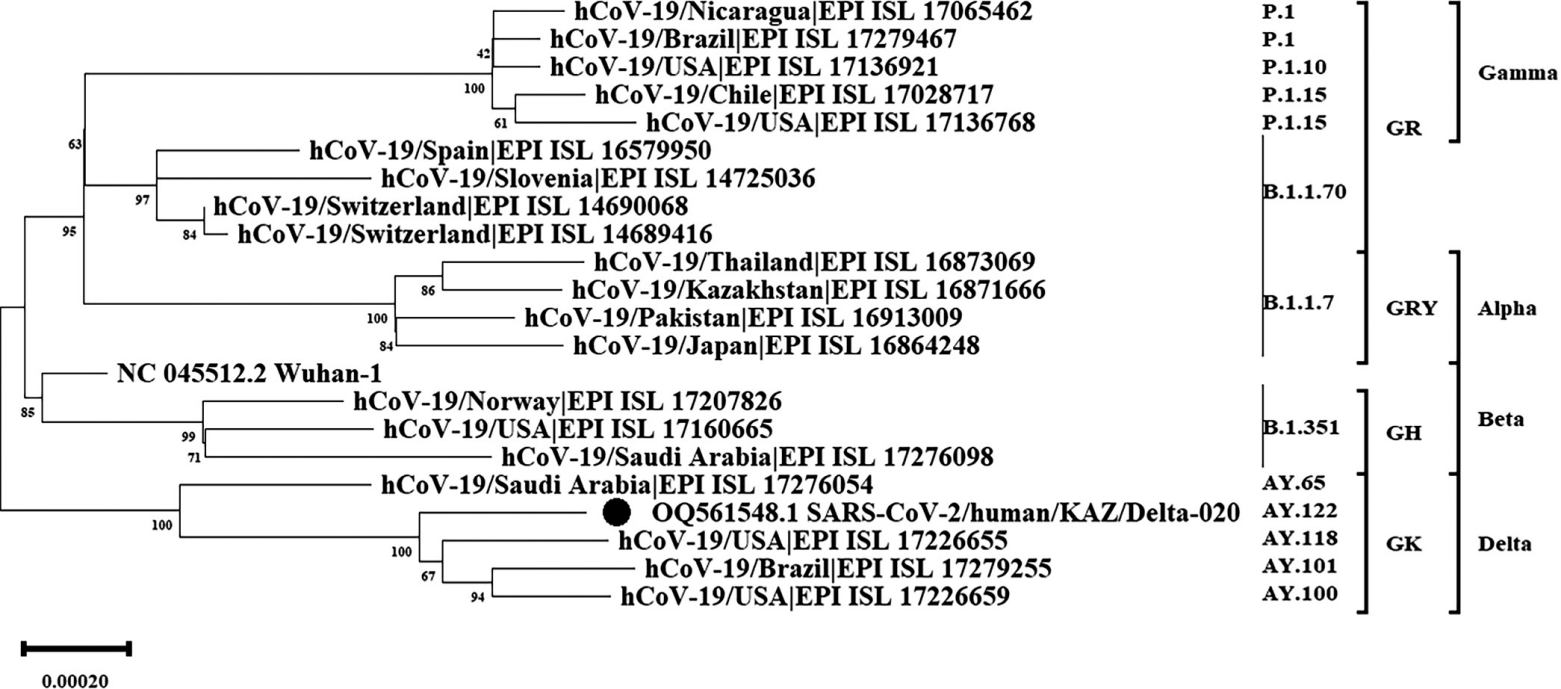
Phylogenetic analysis of strain SARS-CoV-2/Human/KAZ/Delta-020/2021 and 20 global strains belonging to lineages P.1, P1.10, P.1.15, B.1.1.70, B.1.1.7, B.1.351, AY.65, AY.118, AY.101, and AY.100, obtained from GISAID (https://gisaid.org/), and 1 reference strain belonging to lineage B, obtained from NCBI. The phylogeny was inferred using the neighbor-joining method (13). The optimal tree is shown. The percentage of replicate trees in which the associated taxa clustered together in the bootstrap test (1,000 replicates) are shown below the branches (14). The tree is drawn to scale, with branch lengths in the same units as those of the evolutionary distances used to infer the phylogenetic tree. The evolutionary distances were computed using the Tamura-Nei method (15) and are shown as the number of base substitutions per site. This analysis involved 22 nucleotide sequences. The codon positions included were 1st, 2nd, 3rd, and noncoding. All positions containing gaps and missing data were eliminated (complete deletion option). There were a total of 22,107 positions in the final data set. Evolutionary analyses were conducted using MEGA11 (16). Here, the *x* axis represents the scale of the tree.

Phylogenetic analysis showed that the genome of the studied strain belongs to lineage AY.122 (according to the Pangolin database), clade GK (according to the GISAID database), and the Delta variant (according to the WHO nomenclature) (Fig. 1).

### Data availability.

The complete nucleotide sequence of strain SARS-CoV-2/Human/KAZ/Delta-020/2021 was deposited at GenBank under the accession number OQ561548.1.
